# New Treatment for Aphonia

**Published:** 1870-10

**Authors:** 


New Treatment for Aphonia.—Dr. H. K. Olliver, of Boston, contributes a paper to the *Am. Jour. of Med. Sciences*, in which he describes a novel mode of treatment for "Phonic Paralysis," or "paralysis of the adductors of the vocal cords," a form bilateral in character, and usually dependent on debility, hysteria, or some other impairment of the nervous system, but sometimes upon emotional influences and weakness of the muscles following laryngitis or straining of the voice. The laryngoscope reveals partial or complete paralysis of the cords. In the latter case, these ligaments remain widely open on attempted phonation, while in the former, they approach the median line, but not sufficiently to produce a sound, or if they do, are not held together with sufficient force for this purpose. The methods of treatment commonly employed in this affection have been galvanism, stimulants to the larynx, the "gymnastics of the larynx," the use of anæsthetics and general hygiene. Moreover, the voice has been known to be restored suddenly, under some general excitement or mental impression.

In addition to the above treatment, he proposes that by the manipulation of the larynx. This consists chiefly in compression of the thyroid cartilages in their posterior and upper part, by the thumb and forefinger. Such compression practiced on the dead subject will approximate the arytenoid cartilages and the vocal cords, which latter will become tense. In the living subject, pressure produces the same effect, as may be seen by the laryngoscope.

In using this treatment, the patient is instructed to make an attempt to produce a sound like that of *a* or *ah*, or any sound of the voice whatever, while the larynx is being compressed.

Many of these cases seem to depend on a lack of power to *start the machinery* of the vocal apparatus; when once started, the power of keeping up the action is easily afforded; the paralysis seems to be of an unusual character. Well-known instances of sudden restoration of voice show a condition peculiar to the laryngeal muscles. Quacks have restored the voice immediately by grasping the throat and violently compressing it. Such recoveries may have been owing partly to a mental impression upon the patient, but may have been aided by such an adaptation of the parts of the larynx as is described, albeit a condition unappreciated by the operator.

As an aid to this manipulation he takes advantage of the valvular nature of the vocal cords. The vocal cords, when approximated, constitute a valve opening outward, while the false cords form a valve opening inward. In the complete glottic closure, when inspiration and expiration are impossible, the former act is prevented by the vocal cords and the latter by the false cords. The degree of approximation necessary for producing vibration with inspiration, seems to be considerably less than that required with expiration, proof that the form of the cords offers a greater resistance to the inflowing than the outflowing air. Now, by the method suggested, the cords are sufficiently approximated to offer resistance to the air by compression of the cartilage, and a still greater approximation sufficient for vibration of these ligaments, is made by strong and sudden inspiration. In the two most obstinate cases reported, a voice sound could be produced by inspiration before any could be made by expiration. When once started, however, the natural voice was soon established. Dr. O. gives the history of six cases—all in which he has employed this method of treatment—and they all show successful results. The longest time any one had been aphonic was eight years, and the shortest three months.

The manipulation and pressure were made for a few minutes at a time, intervals of rest being allowed between, and no case required a treatment in this way for more than an hour. In nearly all the cases there was a tendency to whisper after the voice was restored, and the patients were instructed to read aloud daily. This had the effect to make the cure complete.

